# Effects of early hemodynamic resuscitation on left ventricular performance and microcirculatory function during endotoxic shock

**DOI:** 10.1186/s40635-015-0049-y

**Published:** 2015-05-08

**Authors:** Alejandra López, Juan Carlos Grignola, Martín Angulo, Ignacio Alvez, Nicolás Nin, Gonzalo Lacuesta, Manuel Baz, Pablo Cardinal, Ivana Prestes, Juan P Bouchacourt, Juan Riva, Can Ince, Francisco Javier Hurtado

**Affiliations:** Pathophysiology Department, University Hospital, School of Medicine, Universidad de la República, Av. Italia 2870, 15th Floor, CP 11600 Montevideo, Uruguay; Anesthesiology Department, University Hospital, School of Medicine, Universidad de la República, Av. Italia 2870, 15th Floor, CP 11600 Montevideo, Uruguay; Department of Intensive Care Adults, Erasmus MC University Medical Center, ‘s-Gravendijkwal 230, 3015 CE Rotterdam, The Netherlands

**Keywords:** Endotoxic shock, Early hemodynamic resuscitation, Microcirculation, Video microscopy, Left ventricular function

## Abstract

**Background:**

Microcirculation and macrohemodynamics are severely compromised during septic shock. However, the relationship between these two compartments needs to be further investigated. We hypothesized that early resuscitation restores left ventricular (LV) performance and microcirculatory function but fails to prevent metabolic disorders. We studied the effects of an early resuscitation protocol (ERP) on LV pressure/volume loops-derived parameters, sublingual microcirculation, and metabolic alterations during endotoxic shock.

**Methods:**

Twenty-five pigs were randomized into three groups: LPS group: *Escherichia coli* lipopolysaccharide (LPS); ERP group: LPS + ERP based on volume expansion, dobutamine, and noradrenaline infusion; Sham group. LV pressure/volume-derived parameters, systemic hemodynamics, sublingual microcirculation, and metabolic profile were assessed at baseline and after completing the resuscitation protocol.

**Results:**

LPS significantly decreased LV end-diastolic volume, myocardial contractility, stroke work, and cardiac index (CI). Early resuscitation preserved preload, and myocardial contractility, increased CI and heart rate (*p* < .05). LPS severely diminished sublingual microvascular flow index (MFI), perfused vascular density (PVD), and the proportion of perfused vessels (PPV), while increased the heterogeneity flow index (HFI) (*p* < .05). Despite MFI was relatively preserved, MVD, PVD, and HFI were significantly impaired after resuscitation (*p* < .05). The macro- and microcirculatory changes were associated with increased lactic acidosis and mixed venous O_2_ saturation when compared to baseline values (*p* < .05). The scatter plot between mean arterial pressure (MAP) and MFI showed a biphasic relationship, suggesting that the values were within the limits of microvascular autoregulation when MAP was above 71 ± 6 mm Hg (*R*^2^ = 0.63).

**Conclusions:**

Early hemodynamic resuscitation was effective to restore macrohemodynamia and myocardial contractility. Despite MAP and MFI were relatively preserved, the persistent microvascular dysfunction could explain metabolic disorders. The relationship between micro- and systemic hemodynamia and their impact on cellular function and metabolism needs to be further studied during endotoxic shock.

**Electronic supplementary material:**

The online version of this article (doi:10.1186/s40635-015-0049-y) contains supplementary material, which is available to authorized users.

## Background

Sepsis prevalence continues to increase worldwide with septic shock and multiple organ failure being the main causes of death in critically ill patients [1-3]. Tissue perfusion has been found to be affected early during the course of the septic insult and is secondary to very complex phenomenon that involves left ventricular (LV) dysfunction, systemic hemodynamic alterations, and microcirculatory failure [4-8]. Early goal-directed therapy (EGDT) based on well-defined systemic targets has been used for more than 10 years to guide resuscitation in these patients [9,10]. However, recent multicenter clinical studies have not shown significant differences in outcome when EGDT was compared to other protocol-based resuscitation strategy or even to usual care [11,12]. Hence, the physiological value and clinical impact of commonly used specific targets, and the relationship between macro- and microhemodynamia, remain under discussion.

The mechanisms of LV dysfunction and the particular role of the major determinants of cardiac index (CI) have been extensively studied during septic shock [8,13,14]. Severe LV preload, afterload, myocardial contractility alterations, and maldistribution of blood flow have been described. Moreover, the final hemodynamic profile can be modulated by the quantity, quality, and timing of hemodynamic resuscitation [5].

Microcirculatory dysfunction has been documented in the early phase of sepsis and its severity has been related to poor outcome [6,7,15,16]. Septic shock appears to cause vascular dysregulation making tissue perfusion dependent on blood pressure. Furthermore, microvascular perfusion could be disrupted by circulating inflammatory mediators that directly damage the peripheral vascular bed. To maintain organ perfusion, current guidelines recommend a mean arterial pressure ≥65 mm Hg based on the infusion of fluids, and vasoactive and inotropic drugs [9,10]. However, this cutoff value is also under discussion [17,18]. Besides the fact that delayed vasopressor/inotropic therapy is associated with an increased risk in mortality [10], the effectiveness of hemodynamic resuscitation depends on many other factors such as the timing of interventions, the stage of sepsis, and presence of comorbidities.

Since tissue hypoperfusion leads to increased blood lactate and decreased mixed venous oxygen saturation (SvO_2_) values, these variables have been used to monitor the systemic metabolic dysfunction. However, in the context of sepsis, the mechanisms of these alterations could be multifactorial. Furthermore, high lactatemia and increased SvO_2_ values have also been associated to poor outcome [19,20].

We hypothesized that early resuscitation may restore CO determinants and microcirculatory parameters but could fail to prevent metabolic disorders during endotoxic shock. The objective of the present study was to evaluate the effects of an early resuscitation protocol (ERP) on LV function and systemic hemodynamics and to correlate these findings with sublingual microcirculation and metabolic alterations in an animal model of endotoxic shock.

## Methods

The study was conducted at the Experimental Laboratory of the Department of Pathophysiology, School of Medicine, Universidad de la República. The experimental study was approved by the Honorary Committee for Animal Research, School of Medicine (CHEA # 071140-000310-07; www.urbe.fmed.edu.uy/). The Institutional Committee is under the regulations of the National Committee for Animal Research (CNEA; http://www.cnea.org.uy/).

### Animal preparation

Twenty-five female Duroc-Pampa pigs, with mean weight of 24.3 ± 4.1 kg, were studied. All the animals received ketamine (5 mg/kg, i.m.) followed by thiopental (10 mg/kg, i.v.), and fentanyl (0.01 mg/kg, i.v.). Anesthesia was maintained by continuous thiopental (5 mg/kg/h) and fentanyl (0.02 mg/kg/h) infusions. After systemic and local anesthesia (lidocaine 1%), the animals were tracheostomized and mechanically ventilated (Amadeus Hamilton Medical AG, Rhäzüns, Switzerland). Volume-controlled ventilation was provided under neuromuscular blockade with atracurium (0.6 mg/kg/h, i.v.) continuous infusion. The level of anesthesia and neuromuscular blockade was controlled by recording arterial pressure, heart rate, and respiratory efforts in response to nociceptive stimuli. Additional anesthesia or analgesia was administered when necessary, according to monitoring. Tidal volume and positive end expiratory pressure (PEEP) were set at 8 mL/kg and 5 cm H_2_O respectively. The respiratory rate was adjusted to maintain an arterial carbon dioxide partial pressure (PaCO_2_) between 40 and 45 mm Hg. Body temperature was kept stable between 36.5°C and 37.5°C using a heating pad. After anesthesia, the right femoral artery and vein were catheterized. Systemic arterial pressure was measured continuously with a solid state catheter (Millar model SPC-370, 7F, 120 cm, Millar Instruments Inc., Houston, TX, USA). A pulmonary artery balloon catheter was placed to measure central venous pressure (CVP), pulmonary artery pressure (PAP), and pulmonary arterial occlusion pressure (PAOP). SvO_2_ was also obtained from this catheter. CO was measured by the thermodilution technique (Oximetrix 3 Computer, Abbott, Chicago, IL, USA), and CI was calculated as CO/body weight. Arterial lactate, SvO_2_, and arterial blood gases were measured with a blood gas analyzer (ABL 700 Series, Radiometer, Copenhagen, Denmark). All the blood samples were taken simultaneously and by duplicate to minimize preanalytical errors. The mean value of these samples was taken for each variable.

The heart was exposed via an anterior sternotomy. An inferior cava vein occlusion pneumatic device was placed for controlled modifications of preload. A 7-Fr, 12-electrode, dual-field, combined pressure-volume (P-V) conductance pigtail catheter (Mikro-tip catheter, model SPC-551, Millar Instruments Inc., Houston, TX, USA) was inserted through the right carotid artery and advanced to the LV apex for the assessment of LV P-V relationships. The correct position of the conductance catheter was confirmed by monitoring individual segmental P-V loops. After completing the experiments, the animals were euthanized under deep anesthesia with an overdose of potassium chloride.

### Experimental protocol

After surgery, a 30-min stabilization period was allowed before baseline measurements. Then simultaneous recordings of systemic hemodynamics, sublingual microcirculatory video microscopy, and metabolic parameters were obtained in all the animals (T0). A representative example of hemodynamic monitoring is presented in the Additional file 1: Figure 1. After baseline measurements, endotoxic shock was induced by 0.025 mg/kg i/v *Escherichia coli* lipopolysaccharide endotoxin (LPS, serotype 0111:B4, Sigma-Aldrich. St. Louis, MO, USA), given during a 1-h infusion via the femoral vein. Continuous saline solution administration (8 mL/kg/h) was used to prevent hypovolemia secondary to the previous fasting period, the surgical preparation, and baseline metabolic loses. All measurements were repeated after 180 min (T180), once the hemodynamic resuscitation protocol was completed. The animals were randomly assigned to one of three groups:

 LPS group (LPS). These animals received basal saline perfusion but were not treated with any further hemodynamic support after LPS injection.

Early resuscitation protocol group (ERP). The ERP group was treated from the very beginning as follows: a) volume expansion with gelatin solution (Haemacell, 250 mL/h; Sanofi-Aventis, Paris, France) from baseline (T0) and during the first 120 min; b) noradrenaline 0.1 mcg/kg/min started 60 min after LPS; c) dobutamine 10 mcg/kg/min started 120 min after LPS. The vasoactive and inotropic drugs doses were defined from previous pilot experiments. The selected doses were effective to maintain a mean arterial pressure (MAP) between 50 and 60 mm Hg and to increase CI when compared to baseline values.

Sham group (SHAM). These animals only received basal saline solution.

### Ventricular function data acquisition and analysis

All the ventricular, arterial, and pulmonary signals were simultaneously monitored in real time and recorded by a multichannel digital acquisition system (200 Hz, SAMAY MD16, Montevideo, Uruguay). Individual cardiac cycles were identified using the first minimum that preceded the peak of the first derivative of LV pressure calculated digitally. Data were recorded at steady-state conditions and during vena cava occlusion. The P-V catheter was connected to a signal conditioner (Leycom Sigma 5DF, Zoetermeer, Netherlands) in order to record LV volume. LV pressure was measured simultaneously (control unit model TC-510, Millar Instruments Inc., Houston, TX, USA). To improve volume estimation, we used a dual-field excitation method which generates a more homogeneous intracavity electric field [21]. Measured time-dependent LV volume [(*V*(*t*)] was determined using the following equation:

$$ V(t)=1/\alpha .\left[G(t).L2.\rho -\alpha Vp\right] $$

where *G*(*t*) is the instantaneous sum of the conductances from each intraventricular electrode pair, *α* is a dimensionless constant, *L* equals distance between sensing electrodes, *ρ* equals the resistivity of blood which is inversely related to conductivity, and *Vp* is the correcting volume for the conductance of the surrounding tissues (parallel conductance). To determine *Vp*, 10 mL of hypertonic saline (5% NaCl) were injected as a bolus into the right atrium through the left jugular vein, causing a transient increase in measured volume, *G*(*t*), without significantly altering cavity volume. To correct for underestimation of true volume, the results measured by the conductance catheter were compared to the results of an independent measurement of CO made by thermodilution. *ρ* was measured at regular intervals using the CD Leycom resistivity meter.

Global LV function was estimated by LV ejection fraction (LVEF). LV preload was assessed by measuring LV end-diastolic volume (LVEDV). Stroke work (SW) was estimated as the integral of the P-V loop. Preload recruitable stroke work (PRSW) was used to estimate LV myocardial contractility [22]. Eight to ten successive beats were selected from the P-V loops measured during vena cava occlusion. A simple linear regression equation was fit to the relationship of SW and end-diastolic volume obtained during vena cava occlusion. This slope describes the PRSW. Among other measurements of LV contractility, this index has been shown to be the most reproducible and relatively independent of the effects of preload and afterload [22,23].

### Microcirculation analysis

The microcirculatory network was evaluated in the sublingual mucosa of the animals with the sidestream dark field (SDF) imaging device with a 5× objective (Microscan®, MicroVision Medical, Amsterdam, Netherlands). Different cautions were followed to obtain adequate quality images and to ensure good reproducibility. After saliva removal, steady video images of at least 10 s were obtained while avoiding pressure artifacts. The video clips were stored as AVI files to allow computerized frame-by-frame image analysis. SDF images were acquired from at least three different sublingual places. For each measurement, the average of three videos was taken. Adequate focus and contrast adjustment were verified. The entire sequence was used to calculate the characteristics of microvascular blood flow, particularly the presence of stopped or intermittent flow. Video clips were blindly and randomly processed by the same investigator using a semi-quantitative analysis [24-26]. Each image was divided into four equal quadrants, and small vessels (<20 μm diameter) were identified. Microvascular density (MVD) was determined as the number of vessels per mm^2^. Flow quantification was performed using a score that distinguishes between no flow (0), intermittent flow (1), sluggish flow (2), and continuous flow (3), assigning a value to each individual vessel. The overall score, i.e., the microvascular flow index (MFI), corresponds to the average of all the individual vessels. The final MFI was obtained from the average of 12 quadrants (three videos, four quadrants each). To determine perfusion heterogeneity in each territory, the heterogeneity flow index (HFI) was calculated as the highest MFI minus the lowest MFI divided by the mean MFI. The proportion of perfused vessels (PPV) was calculated as ((Total number of vessels − Number of vessels with flow = 0 or 1)/Total number of vessels) × 100. Finally, we calculated the perfused vessel density (PVD) as MVD × PPV.

### Statistical analysis

The normal distribution of all the physiological variables was controlled by the Shapiro-Wilk test. Accordingly, data are shown as mean ± SD. Paired *t*-test was used to analyze changes over time in each group (T0 vs T180). One-way ANOVA with Bonferroni correction was used to analyze the differences between the three groups at each time point (SPSS Statistical Package, version 17.0). We used a piecewise linear function with two segments to study the correlation between MFI and MAP. The coefficients of the bilinear curves were computed by a least-square nonlinear fitting routine using the Levenberg-Marquardt procedure (OriginLab, version 9.1.0). Differences were considered statistically significant when *p* < .05.

## Results

### Systemic hemodynamics

MAP was significantly decreased in the LPS and ERP groups when compared to baseline (*p* < .05). However, the final MAP values were higher in the ERP animals when compared to LPS group (*p* < .05). CVP and PAOP did not show significant changes. Early resuscitation significantly increased heart rate (*p* < .05). LPS administration was followed by a significant decrease in CI (*p* < .05). In contrast, CI was increased above baseline in the ERP group, and this value was significantly higher when compared to nonresuscitated animals (*p* < .05). Systemic vascular resistance index (SVRI) was decreased below baseline in the LPS and ERP groups (*p* < .05), while nonsignificant changes were observed in the Sham group.

### LV function

**Figure 1:**
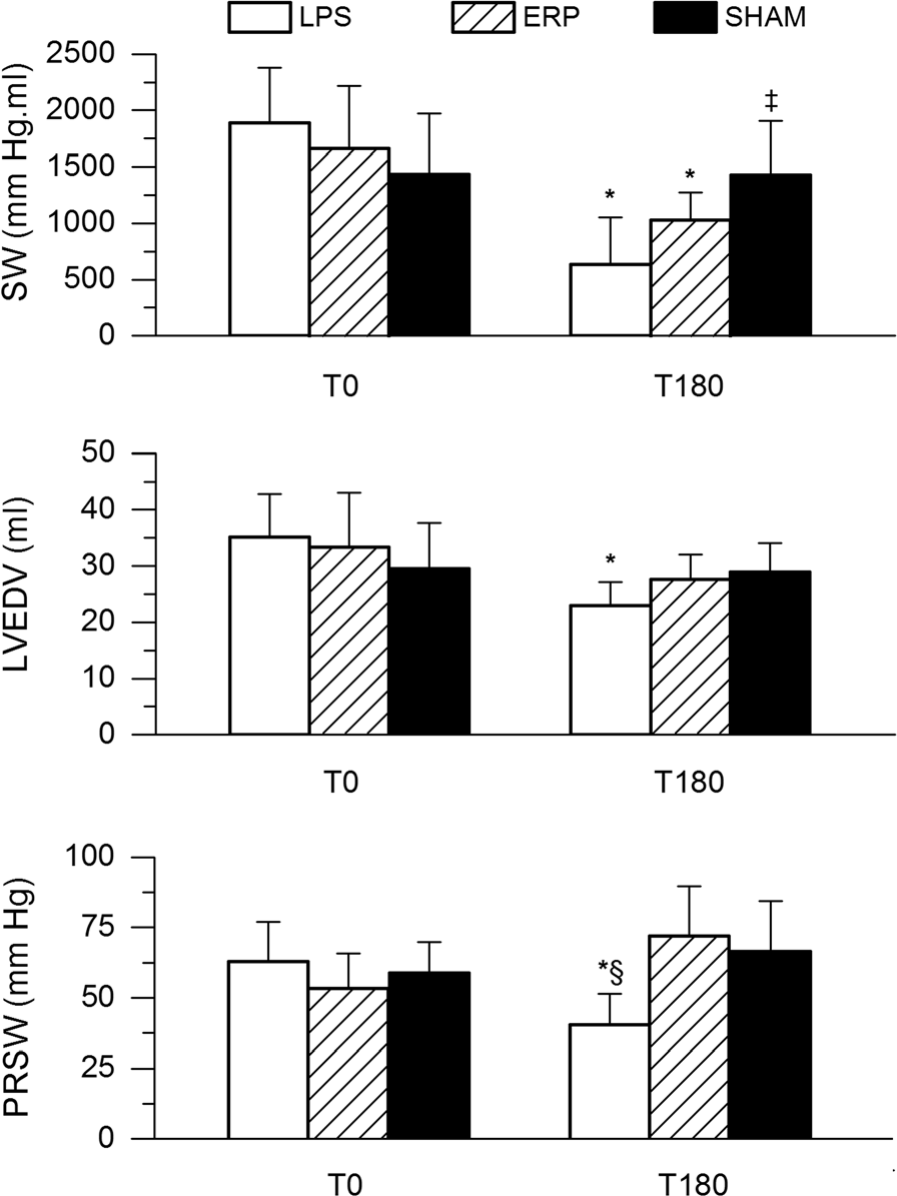
**LV function parameters.** LPS group (white bars), ERP group (gray bars), and Sham group (black bars). T0, baseline T180; final values (mean ± SD). SW, stroke work; LVEDV, left ventricular end-diastolic volume; PRSW, preload recruitable stroke work. *p < .05 compared to baseline values; ‡*p* < .05 when compared to LPS group; §*p* < .05 when compared to other groups.

The main LV function parameters are shown in Figure 1 for the different groups. A decrease in LV end-diastolic volume (LVEDV) was observed in the LPS group, with LVEDV being reduced from 35.2 ± 7.6 to 23 ± 4.1 mL (*p* < .05). Neither ERP nor Sham groups showed significant LVEDV changes. SW was significantly decreased in both, LPS (1,891 ± 491 to 635 ± 415 mm Hg/mL), and ERP (1,669 ± 558 to 1,037 ± 245 mm Hg/mL) groups (*p* < .05). Of note, the magnitude of SW reduction in the ERP group was much lower than the one observed in the LPS animals (34% vs 62%, *p* < .05).

LV myocardial contractility evaluated by the PRSW was reduced after LPS administration (62.9 ± 14.2 to 40.4 ± 11.1 mm Hg, *p* < .05). This value was significantly lower when compared to the other groups (p < .05). PRSW showed a nonsignificant change from 53.7 ± 12.4 to 72.2 ± 17.7 mm Hg above baseline after resuscitation. Myocardial contractility remained without significant changes in the Sham group. Representative LV P-V loops showing the family loops recorded during inferior cava vein occlusion, at baseline and at the end of the experiments in the LPS group, are presented in the Additional file 2: Figure 2.

### Systemic oxygen metabolism and metabolic parameters

Data is shown in Table 1. Systemic O_2_ delivery index (IDO_2_) baseline values remained stable in the Sham animals but decreased in the LPS group (*p* < .05) and significantly increased in the ERP animals (*p* < .05). This final value was significantly higher when compared to baseline and to the LPS group.

**Table 1:** Systemic hemodynamics and metabolic data

**Variables**	**Time**	**LPS group (** ***n*** **= 9)**	**ERP group (** ***n*** **= 8)**	**SHAM group (** ***n*** **= 8)**
MAP (mm Hg)	T0	92.3 ± 20.1	85.7 ± 22.1	86.4 ± 18.1
	T180	38.1 ± 16.2 *	57.8 ± 10.8 * §	84.2 ± 12 §
CVP (mm Hg)	T0	7.1 ± 3.5	4.8 ± 2.8	5.1 ± 2.1
	T180	6.5 ± 2.9	7.1 ± 2.3	7 ± 2.1
PAOP (mm Hg)	T0	5.6 ± 2.7	5.3 ± 2.4	5.5 ± 4.5
	T180	6.4 ± 2.7	6.9 ± 3.4	6.5 ± 4
HR (bpm)	T0	97 ± 18	100 ± 24	98 ± 19
	T180	106 ± 40	157 ± 21 * §	95 ± 25
CI (mL/min/kg)	T0	87.6 ± 29.6	69.4 ± 20.5	65.3 ± 16.7
	T180	45 ± 22.6 *	95.8 ± 36.6 * ‡	65.1 ± 25.2
SVRI (dyn.s/cm^5^)/kg	T0	163.9 ± 96.2	166 ± 57	209.3 ± 91
	T180	108.1 ± 63.4 *	76.2 ± 22.6 *	201.8 ± 71.4 §
IDO_2_ (mL/min/kg)	T0	13.4 ± 5.4	11 ± 2.8	9.1 ± 4.7
	T180	6.4 ± 3.6 *	13.3 ± 4.2 * ‡	10 ± 4
IVO_2_ (mL/min/kg)	T0	6.2 ± 1.8	6.4 ± 1.3	5.7 ± 2.8
	T180	3.8 ± 1.5 *	5.9 ± 2.2	5.3 ± 2.1
ERO_2_ (%)	T0	54.5 ± 10.2	58.6 ± 5.7	52.4 ± 13.4
	T180	65.2 ± 13.5 *	44.5 ± 8.8 * ‡	54 ± 6
Lactate (mmoL/L)	T0	1.3 ± 0.4	1.4 ± 0.6	1.4 ± 0.6
	T180	5.1 ± 2.4 *	4.1 ± 2.7 *	1.1 ± 0.3 §
SvO_2_ (%)	T0	48.8 ± 8.4	41.7 ± 7.1	49 ± 13.2
	T180	34 ± 14.4 *	55.4 ± 9.3 * ‡	46.6 ± 8.7

Values are mean ± SD. LPS, *E. coli* lipopolysaccharide; ERP, early resuscitation protocol; MAP, mean arterial pressure; CVP, central venous pressure; PAOP, pulmonary artery occlusion pressure; HR, heart rate; CI, cardiac index; SVRI, systemic vascular resistance index; IDO_2_, systemic oxygen delivery index; IVO_2_, systemic oxygen consumption index; ERO_2_, systemic oxygen extraction ratio; Lactate, arterial lactate; SvO_2_, mixed venous oxygen saturation. * *p* < .05 compared to baseline values; ‡*p* < .05 compared to LPS; §*p* < .05 compared to other groups.

Lower systemic O_2_ consumption index (IVO_2_) values were observed in the LPS animals when compared to baseline (*p* < .05). However, no significant statistical changes were registered in the other study groups. Oxygen extraction ratio (ERO_2_) was increased in the LPS group (*p* < .05) and decreased significantly after resuscitation (*p* < .05). SvO_2_ decreased in the LPS group and increased significantly when compared to baseline after resuscitation (*p* < .05). No changes were observed in the Sham animals.

As shown in Table 1, baseline arterial lactate concentration values were normal in all the groups and remained stable in the Sham animals by the end of the experiments. Lactate significantly increased in both the LPS and ERP groups by the end of the experimental period (*p* < .05), with no significant difference between these two values.

### Sublingual microcirculation

Sublingual microcirculation parameters of the small vessels are presented in Table 2. The MVD was reduced in the ERP animals at the end of the experiments (*p* < .05). This parameter did not show any significant change in the other groups. The PVD decreased significantly in both the LPS and ERP groups when compared to baseline and to the Sham animals (*p* < .05). Small vessels MFI decreased significantly by the end of the experiments in the LPS animals (*p* < .05), and this value was significantly lower when compared to the final MFI measured in the other groups (*p* < .05). At the same time, MFI was not different in the ERP animals when compared to the Sham group. HFI increased both in the LPS and ERP groups by the end of the experimental period (*p* < .05). The final HFI in LPS was significantly higher than in ERP and Sham groups. No changes in HFI were documented in the Sham group. PPV significantly decreased after LPS (*p* < .05), increased in Sham animals, and showed a no significant changes after resuscitation.

**Table 2:** **Sublingual microcirculatory parameters**

**Variables**	**Time**	**LPS group (** ***n*** **= 9)**	**ERP group (** ***n*** **= 8)**	**SHAM group (** ***n*** **= 8)**
MVD (vessels/mm)	T0	31.1 ± 9.4	35.6 ± 9.5	24.7 ± 4.9
	T180	25.4 ± 6.8	23.1 ± 5.1 *	28.3 ± 6
PVD (vessels/mm)	T0	28.9 ± 7.9	33.6 ± 10.4	23.8 ± 3.8
	T180	12.6 ± 8.7 *	16.8 ± 7.2 *	27.4 ± 5.5 §
MFI	T0	2.7 ± 0.28	2.7 ± 0.22	2.6 ± 0.4
	T180	1.1 ± 0.6 * §	2.3 ± 0.45	2.8 ± 0.25
HFI	T0	0.11 ± 0.07	0.16 ± 0.12	0.3 ± 0.25
	T180	1.1 ± 0.4 * §	0.49 ± 0.25 *	0.23 ± 0.16
PPV (%)	T0	93.6 ± 5.2	93.6 ± 6.2	92.4 ± 2.9
	T180	47.5 ± 24.6 *	71.3 ± 27.8	96.9 ± 2.3 * ‡

Values are mean ± SD. LPS, *E. coli* lipopolysaccharide; ERP, early resuscitation protocol; MVD, microvascular density; PVD, perfused vascular density; MFI, microvascular flow index; HFI, heterogeneity flow index; PPV, proportion of perfused vessels (%). * *p* < .05 compared to baseline values; ‡*p* < .05 compared to LPS; §*p* < .05 compared to other groups.

**Figure 2:**
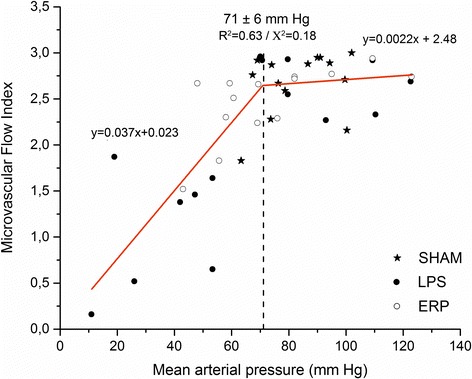
**Correlation between MAP and MFI.** The calculated inflection point of the biphasic relationship for MAP was 71 ± 6 mm Hg (*R* ^2^ = 0.63).

Figure 2 represents the scatter plot between MAP and MFI. The bilinear fitting of the data is represented. The observed biphasic relationship shows that MFI does not correlate with MAP above 71 ± 6 mm Hg. However, microvascular flow severely decreases below this value (*R*^2^ = 0.63). From the same equations, a MFI = 2.63 was calculated for the referred MAP inflection point.

Representative sublingual video microscopy videos at baseline and at the end of the experimental period in the LPS and ERP groups are presented in the Additional file 3: Video 1 and Additional file 4: Video 2.

## Discussion

The targets and monitoring parameters recommended to guide resuscitation of septic shock patients have recently been questioned by emerging clinical studies [11,12]. In this context, the present investigation allowed us to integrate the physiologic behavior of central and peripheral cardiovascular compartments with metabolic alterations in an experimental animal model. To our knowledge, the simultaneous assessment of LV pressure/volume parameters, along with sublingual microcirculation has not been performed before in these experimental conditions.

LPS induced an acute and severe hypodynamic shock state, leading to generalized tissue hypoperfusion and oxygen deprivation. As observed from the LV P-V loop analysis, cardiac output (CO) and arterial pressure decreased following acute onset of hypovolemia, depressed myocardial contractility, and decreased systemic vascular resistances. Under these conditions, we registered the simultaneous compromise of sublingual microcirculation. Then, the accompanying metabolic abnormalities should be attributable to generalized tissue hypoperfusion. The relevance of the disruption of the microcirculation has been reported during septic shock in the clinical setting. It has also been shown that the severity and persistence of these alterations correlate with poor outcome [15,16]. Furthermore, it has been demonstrated that tissue perfusion recovery may ameliorate the inflammatory response, improve metabolic disorders, and prevent multiple organ failure. Thus, the importance of early resuscitation has been remarked by several studies [15,27,28].

The role and the mechanisms of decreased myocardial contractility during sepsis have been profoundly studied [8,13,14]. We found that early resuscitation was effective to preserve myocardial contractility and restore LV preload. These factors contributed to change macrohemodynamics from an initial hypodynamic to a hyperdynamic shock state. Thus, increased CI and oxygen delivery were the result of the combined effects of volume administration, noradrenaline, and dobutamine as proposed by the protocol applied in this study. On the other hand, it has been described that aggressive resuscitation therapies may increase morbidity and mortality, probably due to excessive volume administration, or to a deleterious effect of inotropic or vasoactive drugs. In fact, the increased heart rate observed in the ERP group could be the expression of a non-desirable chronotropic effect of dobutamine [29,30].

Since the introduction of hand-held video microscopy, the relationship between systemic hemodynamic and sublingual microcirculatory parameters has been studied in different clinical conditions and in response to some therapeutic interventions [31-37]. A lack of correlation and great individual variability between variables such as MAP and MFI have been communicated from these studies [31,34,38]. The vasoconstrictor effect of noradrenaline may increase arterial pressure independently of microvascular flow changes [33,34]. When the correlation between MAP and MFI was analyzed in our study, a biphasic relationship was found. From these data, the inflection point that separates both slopes could represent the limits of microvascular autoregulation mechanisms. Thus, reaching MAP values higher than 71 ± 6 mm Hg could have no beneficial effect on tissue perfusion. On the other hand, a proportional decrease in MFI will result when MAP decreases below this value. Consistent with previous reports, the MFI calculated from the equations for this inflection point was 2.63. Almost the same MFI has been defined by other authors as the cutoff value that separates normal from decreased microvascular perfusion. Pranskunas et al. showed that 66% of patients with clinical signs of impaired organ perfusion had MFI lower than 2.6. These authors defined this cutoff value as the lower bound of the 95% CI of MFI in a normal population [39]. Other authors proposed a similar value to determine the incidence of microcirculatory alterations in critically ill patients. Thus, Vellinga et al. in a multicenter clinical study found that 17% of the critically ill patients had MFI values lower than 2.6. Interestingly, in patients with tachycardia, an abnormal MFI was independently associated with an increased risk of hospital death [40].

Despite these considerations, it has been emphasized that the MFI only represents the convective component of tissue perfusion and oxygenation. To further characterize microcirculatory disorders, other parameters need to be analyzed. Because PVD takes into account both MVD and MFI changes, it represents also the diffusive component of tissue oxygenation, resulting in a more integrative variable. Finally, the distribution of microvascular blood flow should be taken into account and is represented by the HFI [24].

As shown in our LPS animals, tissue perfusion was characterized by a severe decrease of MFI and PVD concluding that all the determinants of tissue oxygenation were compromised. At the same time, an increased heterogeneity of microvascular flow distribution was also documented.

When microcirculation was analyzed during the hyperdynamic endotoxic shock conditions, MFI was found to be relatively preserved. On the other hand, the decreased MVD observed after resuscitation should be interpreted with caution, since it could be attributed to the effect of tissue edema secondary to volume administration, rather than a real decrease in the number of small vessels. However, the observed compromise of the PVD and the increased microvascular heterogeneity found in these animals lead us to conclude that the systemic effects of the resuscitation protocol were not accompanied by a significant restoration of the microcirculatory parameters.

Whether a hyperdynamic microvascular flow occurs during septic shock has been studied in clinical series. Dubin et al. measuring capillary red blood cell (RBC) velocity did not find any increase in this parameter in septic shock patients [25]. However, Konning et al. found that cardiac surgery patients during and after cardiopulmonary bypass showed systemic and microvascular hyperdynamic changes defined by increased CI, decreased systemic vascular resistances, and increased capillary RBC velocity. Interestingly, this pattern was associated with increased microvascular heterogeneity and decreased oxygen extraction capacity. The authors concluded that the observed microvascular abnormalities may induce a peripheral shunting of oxygen that may explain the decreased arteriovenous oxygen gradient [41]. Since we did not measure RBC velocity, we cannot speculate about the occurrence of hyperdynamic conditions following the systemic effects of resuscitation.

Finally, the persistent high arterial lactate and the increased SvO_2_ values when compared to baseline and to the LPS animals could be explained by the observed microcirculatory alterations. Despite the mechanisms that generate the metabolic disorders are complex and multi factorial, they could be attributed to peripheral shunting, leading to decreased oxygen extraction in a heterogeneous microcirculatory compartment [7,42,43]. Furthermore, the persistence of these alterations may announce progression to multiple organ failure and poor outcome.

Whether the observed microvascular dysfunction could also be associated with cellular or mitochondrial alterations cannot be ruled out from this study [44].

### Limitations of the study

The resuscitation protocol used fixed infusion rates for dobutamine and noradrenaline. Our experimental model was not designed to titrate different volume, noradrenaline, or dobutamine doses. Thus, we cannot speculate about how different doses could affect tissue perfusion. From previous pilot experiments testing different LPS doses and resuscitation protocols, it was determined that the employed treatment was able to improve the macrohemodynamic parameters as shown. Other animal models of sepsis or resuscitation protocols could show different impact either on the central or the peripheral hemodynamic compartments.

Sublingual microcirculation may not represent the perfusion of other territories as the splanchnic area. Dubin et al. showed persistent villi hypoperfusion but sublingual and gut serosa MFI recovery after resuscitation in sheep endotoxemia [45]. This study showed the complexity of the microcirculatory behavior. Thus, data obtained from the sublingual region may not represent other territories.

## Conclusions

This experimental study explored the effects of LPS and hemodynamic resuscitation on LV function and microcirculation. The treatment protocol restored LV preload and myocardial contractility, improving CI and macrohemodynamia. However, although MFI was preserved, major microcirculatory alterations remained after early resuscitation. Although other mechanisms are possible, the persistent metabolic alterations could be explained by the microvascular dysfunction. The inflection point calculated from the biphasic relationship between MAP and MFI could show the limits of microvascular autoregulation. The effects of different hemodynamic resuscitation protocols and their impact on macro- and microhemodynamic variables need to be further investigated.

## Additional files

Additional file 1: Figure S1. Representative cardiovascular and left ventricular (LV) hemodynamic monitoring. AoP, aortic blood pressure; dP/dt, first derivative of LVP; LVP, LV pressure; LVV, LV volume; PAP, pulmonary artery pressure. Measurements are represented at baseline, during LPS infusion (gray rectangle), and at the end of the experimental period. Additional file 2: Figure S2. Representative left ventricular (LV) P-V loop during vena cava occlusion of the LPS group at T0 (A) and T180 (C). Graphic representation of the PRSW at T0 (B) and T180 (D) of the same animal. LVP, LV pressure; LVEDV, LV end-diastolic volume; PRSW, preload recruitable stroke work. Additional file 3: Video S1. Video microscopy in the LPS group. Representative images of microvascular video microscopy recorded at baseline (T0) and at the end of the experimental period after LPS administration (T180). Additional file 4: Video S2. Video microscopy in the ERP group. Representative images of microvascular video microscopy recorded at baseline (T0) and at the end of the experimental period in the early resuscitated animals (T180).

## Abbreviations

CO: cardiac output
CI: cardiac index
CVP: central venous pressure
ERP: early resuscitation protocol
ERO_2_: oxygen extraction ratio
HFI: heterogeneity flow index
IDO_2_: systemic O_2_ delivery index
IVO_2_.: systemic O_2_ consumption index
LPS: *Escherichia coli* endotoxin lipopolysaccharide
LV: left ventricle
LVEDV: left ventricular end-diastolic volume
MAP: mean arterial pressure
MFI: microvascular flow index
MVD: microvascular density
PAP: pulmonary artery pressure
PAOP: pulmonary arterial occlusion pressure
PRSW: preload recruitable stroke work
PPV: proportion of perfused vessels
PVD: perfused vascular density
SDF: sidestream dark field
SvO_2_: mixed venous oxygen saturation
SVRI: systemic vascular resistance index
SW: stroke work.
