# Supplementary figures and images for: Effects of early hemodynamic resuscitation on left ventricular performance and microcirculatory function during endotoxic shock
Source: Intensive Care Med Exp. 2015 May 8;3:14. doi: 10.1186/s40635-015-0049-y (PMC4513023; doi:10.1186/s40635-015-0049-y)

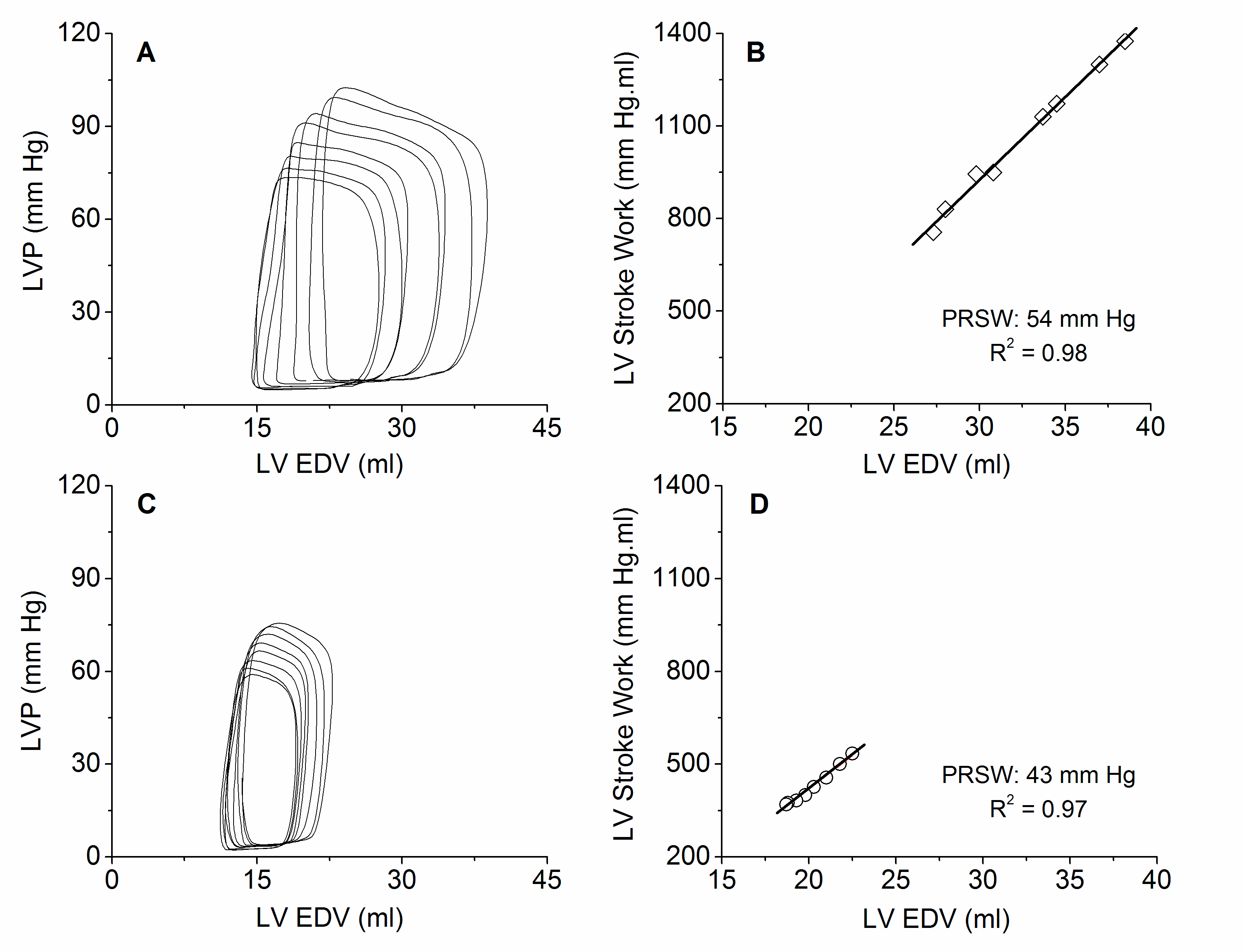

Supplement: Additional file 2: Figure S2. — Representative left ventricular (LV) P-V loop during vena cava occlusion of the LPS group at T0 (A) and T180 (C). Graphic representation of the PRSW at T0 (B) and T180 (D) of the same animal. LVP, LV pressure; LVEDV, LV end-diastolic volume; PRSW, preload recruitable stroke work. [file 40635_2015_49_MOESM2_ESM.tiff]
